# Steroid therapy in septic shock: survey of practice amongst UK critical care physicians

**DOI:** 10.1186/cc12007

**Published:** 2013-03-19

**Authors:** G Rajendran, K Dasari, A Dhrampal

**Affiliations:** 1 Norfolk & Norwich University Hospital, Norwich, UK

## Introduction

Corticosteroid (CS) therapy in sepsis remains controversial and was first introduced in sepsis management for its anti-inflammatory property. CS has found a role in septic shock amelioration with inconsistent outcomes. The Surviving Sepsis Campaign (SSC) includes CS as a Level 2C recommendation in septic shock [1]. Adapting and practicing SSC guidelines vary between critical care units. Accordingly, a survey was conducted to elucidate the usage of CS for septic shock by UK critical care physicians (CCPs).

## Methods

Following approval by the UK Intensive Care Society (ICS), the survey was publicised on the ICS website and its newsletter.

## Results

A total of 81 intensivists responded to this online survey. Seventy-four (92.5%) CCPs prescribed CS only if the septic shock is poorly responsive to fluid resuscitation and vasopressor therapy. Six (7.5%) initiated CS at the same time as vasopressor therapy. None initiated CS for patients with severe sepsis. No CS other than hydrocortisone is being used. The most commonly used intravenous regimen is 50 mg 6 hourly (65%) followed by 50 mg 8 hourly (11%). Only 10% of CCPs would prescribe it by infusion. Less commonly used regimens were 100 mg 8 hourly (6%) and 100 mg 6 hourly (5%). Only 5% would consider adding fludrocortisone. Prior to initiating CS, 5% of CCPs would perform a short synacthen test, while 94% would not. The majority (89%) of CCPs would stop CS after resolution of shock state or when vasopressor infusion is terminated whilst 11% after a fixed duration. Withdrawal of CS also differed, in that 25% tapered/weaned steroids, 31% stopped it abruptly and 44% of CCPs would base their CS cessation pattern on the clinical context. Only 46% of CCPs believe that CS is beneficial whereas 44% were unsure of the benefits in septic shock. Only 29 (36%) responders indicated that their critical care unit had a written protocol for CS in septic shock.

## Conclusion

The perceptions, usage and cessation of CS in septic shock vary but do appear to have shifted in the last decade. A UK survey in 2003 identified that only 60% of ICUs used CS for septic shock and over 22% perform a short synacthen test [2]. It appears that many intensivists are using CS for septic shock, despite conflicting outcome data. We all strive to practice evidence-based medicine but until we have a robust, reliable and methodical randomised control trial that attempts to resolve the CS debate, practice will remain diverse on this subject, as reffected by our survey.
